# A comprehensive lab cookbook for creating carbohydrate modified proteins for therapeutic applications

**DOI:** 10.3389/fphar.2013.00107

**Published:** 2013-09-10

**Authors:** Bibhash Mukhopadhyay

**Affiliations:** Johnson & JohnsonNew Brunswick, NJ, USA

**Keywords:** glycoproteins, analytical techniques, protocol, monoclonal antibody, antibody formation

Glycosylation Engineering of Biophar-maceuticals: Methods and Protocols Edited by Alain Beck is a comprehensive, step by step instruction manual for creating and characterizing therapeutic proteins, in particular, monoclonal antibodies, with carbohydrate modifications.

The book, published under the aegis of Springer Protocols series, is divided into 4 sections:

- “Glycoengineering of therapeutic proteins” focusing on how to produce tailored glycosylated versions of monoclonal antibodies as therapeutic proteins.
- “Glycoanalytics” focusing on analytical characterization involving methods like mass spectroscopy and chromatographic separation to identify and profile signatures of glycosylated proteins.
- “Glycoprotein complex characterization” focusing on analytical methods for epitope mapping and Ag-Ab complexes.
- “PK/PD assays for therapeutic antibodies” focusing on assays to measure specific pharmacological potency readouts for glycosylated therapeutics.

It is obvious from the content that the book is intended as a reference manual of protocols and best practices for the practicing bench scientist. The authors do a marvelous job of covering a wide variety of tools and techniques and bringing them together in one volume. Whereas books have been previously written on the chemistry of glycosylation (see Alexei Demchenko's Handbook of Chemical Glycosylation, focusing on synthesis and structures of glycosylated derivatives), and the biology of protein glycosylation (see Rosalyn Bill's Protein Glycosylation, focusing on purification, function and kinetics of selected glycosylation enzymes and *in vivo* function of glycosylated proteins), this volume is the first comprehensive collection of *HOW* to synthesize glycoproteins *in the lab*.

The organization of chapters in the book has a logical flow with an introduction that sets the reader up for appreciating the salient issues involved in the protocol described subsequently. These sections are followed by those on data interpretation and artifacts and finally ending in notes and references to primary literature.

- The introduction goes into the *WHY* of the experiments i.e., the rationale for the experimental protocol but assumes knowledge of the scientific background. Consequently, the uninitiated reader might have to refer to a standard textbook or review papers to grasp finer detail. This is not a shortcoming of the book because the intended audience is expected to be reasonably well versed with the basics of glycoprotein biology and chemistry before attempting to work in the lab. I believe that these descriptions should have schematic outlines of experimental strategy, ample information that set up an experimentalists' mind to carry out the experiment thoroughly and interpret the results, positive or negative, with appropriate scientific rigor. In this context, I found the description of scientific rationale most comprehensively described in Chapters 4, 5, 6, and 18. Other chapters had them too but these chapters stand-out in the clarity and representation of the experimental rationale and putting it in context of the theoretical knowledge in the glycol-biology space.
- The protocols go into the *HOW* of the experiments. They are very well organized and comprehensive. They provide a step by step recipe for carrying out an experiment or analytical process. Thought I have not tested any of the protocols in the lab, I do believe by reading through them that they are quite comprehensive. At the end of the day, if a reasonably capable experimentalist is able to carry out an experiment for the first time using a protocol, it is considered to be a comprehensive one. Through that filter, I believe that someone wanting to benchmark a process or establish one for the first time will benefit from the descriptions contained in the protocol. They go deeper than what can be gleaned away from the Materials and Methods sections of published papers and hopefully incorporate some of the best practices that came though experience of the authors of the respective chapters.
- The section on data interpretation is not explicitly outlined in every chapter, but sometimes incorporated into the experimental strategy section (e.g., Chapter 5) or sometimes follows the description of the experimental protocols (e.g. Chapters 7, 9, and 19). An experimentalist who is attempting a new technique for the first time will find this section particularly illuminating because s/he can form a mental picture of what the data will look like and how can it be interpreted.
- The notes section is essentially a cheat-sheet, a compilation of experimental best practices. I believe this is an absolutely critical aspect of every chapter because an experimentalist without years of experience (as the authors have) will not necessarily understand the “feel” of the experiment without these best practices.
- Finally, each chapter has a section on primary literature. One shortcoming in this is lack of “loop-back” to the protocol or the notes section. What I would have much appreciated is some form of cross-reference to the notes or the scientific rational/introduction section. In that way, a proficient experimentalist could have gone back to the original reference in a targeted manner, if required.

What was particularly pertinent to me was the focus on monoclonal antibodies as substrate for glycosylation, thereby creating methods that are of therapeutic relevance. Glycoengineered MAb's have gained increasing prominence in the world of targeted drugs, particularly against cancer. Defined glycosylation patterns boost the pharmacological properties of MAb's. It has already yielded Mogamulizumab for refractory Adult T-Cell Leukemia/Lymphoma indication (Anti-CCR4, approved in Japan, commercial under trade name Poteligio) and there are at least 16 other glycol-engineered MAb's in the clinical pipeline including the most advanced Obinutuzumab program from Roche (Phase-III, Anti-CD20 humanized IgG1). Disease indications for which these Glyco-engineered MAb's are being evaluated mostly include hematological malignancy and solid tumor indications in the oncology space, but also disease with immune-modulatory target mechanisms (e.g., Asthma).

This collection of protocols will find an audience with experimentalists in the biopharmaceutical industry as well as various academic labs. Contract research organizations, particularly those focusing on analytical services or synthesis services will also benefit from the protocols described. Finally, the readers should always realize that they are reading in a book form, protocols that are constantly evolving. New techniques, materials and methods are being published every day that improve on or radically change the way we do things. Compilations on protocols are therefore, by definition, to be constantly updated. The publishers and authors can therefore provide a back-up online resource as an adjunct to the desk-top volume for practitioners to use. In absence of that, the shelf-life of a book of protocols is short. I hope the publishers and authors are cognizant of this. Overall, I would strongly recommend this book and advice the authors and publishers to aspire to become the “Maniatis manual” of Antibody Glycobiology.

